# Robust Fine Registration of Multisensor Remote Sensing Images Based on Enhanced Subpixel Phase Correlation

**DOI:** 10.3390/s20154338

**Published:** 2020-08-04

**Authors:** Zhen Ye, Jian Kang, Jing Yao, Wenping Song, Sicong Liu, Xin Luo, Yusheng Xu, Xiaohua Tong

**Affiliations:** 1 College of Surveying and Geo-Informatics, Tongji University, 1239 Siping Road, Shanghai 200092, China; 89_yezhen@tongji.edu.cn (Z.Y.); 1610962@tongji.edu.cn (W.S.); sicong.liu@tongji.edu.cn (S.L.); xinluo_xin@tongji.edu.cn (X.L.); yusheng.xu@tum.de (Y.X.); xhtong@tongji.edu.cn (X.T.); 2 Faculty of Electrical Engineering and Computer Science, Technische Universität Berlin, 10587 Berlin, Germany; 3 School of Mathematics and Statistics, Xi’an Jiaotong University, Xi’an 710049, China; jasonyao@stu.xjtu.edu.cn

**Keywords:** image registration, subpixel matching, phase correlation, multisensor remote sensing images, fine registration

## Abstract

Automatic fine registration of multisensor images plays an essential role in many remote sensing applications. However, it is always a challenging task due to significant radiometric and textural differences. In this paper, an enhanced subpixel phase correlation method is proposed, which embeds phase congruency-based structural representation, *L*_1_-norm-based rank-one matrix approximation with adaptive masking, and stable robust model fitting into the conventional calculation framework in the frequency domain. The aim is to improve the accuracy and robustness of subpixel translation estimation in practical cases. In addition, template matching using the enhanced subpixel phase correlation is integrated to realize reliable fine registration, which is able to extract a sufficient number of well-distributed and high-accuracy tie points and reduce the local misalignment for coarsely coregistered multisensor remote sensing images. Experiments undertaken with images from different satellites and sensors were carried out in two parts: tie point matching and fine registration. The results of qualitative analysis and quantitative comparison with the state-of-the-art area-based and feature-based matching methods demonstrate the effectiveness and reliability of the proposed method for multisensor matching and registration.

## 1. Introduction

Image registration, which is the process of geometrically aligning two or more images of the same scene taken at different conditions, is essential to image analysis tasks involving information extraction from different overlapping images [1]. With the rapid development of sensor technology, remote sensing images have attracted more and more attention due to their increasing spatial and spectral resolution, convenience, and coverage [2]. Remote sensing images from different sensors are able to provide useful complementary information. Multisensor image registration is a fundamental preprocessing step for utilizing these images in a wide variety of applications, such as image fusion, change detection, and environmental monitoring [3,4,5]. However, due to the temporal difference and the diverse properties of sensors or regions in the scene, the image pairs acquired from different optical sensors exist the issues of non-linear intensity differences, textural changes and local distortions [6]. Therefore, automatic registration of multisensor images is a challenging task.

Image registration can be generally divided into coarse registration and fine registration. The coarse registration stage pre-registers the reference and sensed images to eliminate significant rotation and scale differences and shorten the search range through a global transformation model, while the fine registration stage corrects the misalignment and refines the registration performance commonly through a more local or higher-order transformation model [7,8]. Most current remote sensing images are usually attached with georeferencing information that can be employed to remove the obvious geometric differences between images, such as rotation, scale, and global translation [9,10]. In other words, coarse registration of remote sensing images can be achieved by direct georeferencing using sensor models, and the pre-registered image pairs only exist an offset of several or dozens of pixels that require the fine registration stage to compensate. In this study, we focus exclusively on fine registration of remote sensing images.

A typical image registration method consists of two basic steps, i.e., image matching and image warping [11]. The former step extracts and matches the tie points between reference and sensed images that are the distinctive and representative points of the investigated scenes, while the latter step estimates a transformation model from the set of corresponding tie points and then transforms the sensed image to the reference image using image resampling. In order to realize precise and reliable fine registration of multisensor remote sensing images, the image matching part that determines the correspondence relationship of the tie points plays the most crucial role. In the literature, there are two major types of image matching methods: feature-based methods and area-based methods [1,12,13]. The feature-based methods match the features detected separately from each image based on their spatial structure or distance of invariant descriptor vectors. The most widely used local invariant features applied in image registration are the scale-invariant feature transform (SIFT) feature and its variants [14,15,16,17]. However, one of the main limitations of feature-based methods is that they require a sufficient number of highly repeatable features extracted from both images, which is especially difficult in the multisensor cases with obvious radiometric and textural changes.

In contrast, area-based methods rely on the similarity measure directly calculated from the intensity in the corresponding window pairs or even the entire images, which usually outperform feature-based methods in the aspect of precision, distribution, and robustness [18]. These merits enable the area-based methods more effective in fine registration of multisensor remote sensing images [19]. Phase correlation (PC) is an area-based matching technique according to the image information and operation in the frequency domain. By means of fast Fourier transform (FT) and phase information, PC can achieve outstanding performance in theoretical accuracy, computational efficiency, and robustness against the frequency-dependent noise and illumination changes [20]. These merits make it quite feasible for multisensor image registration. When used in coarse registration, PC can be extended to deal with rotation and scale estimation without the need for initialization and iteration using the Fourier-Mellin transform [21,22,23]. For fine registration, PC can be adopted in local template matching even pointwise dense matching with subpixel estimation. Additional operations that ensure the best approximation of the theoretical phase difference model play an important role in the subpixel PC methods. In this study, an enhanced subpixel PC method calculated in the frequency domain is proposed. Three additional operations are embedded into the conventional line fitting-based PC method to improve the practical performance of tie point matching: (1) phase congruency information is adopted as feature representations to reduce the influence of nonlinear intensity differences in multisensor cases; (2) a *L*_1_-norm-based robust low-rank matrix factorization algorithm is used with effective frequency masking to find the best rank-one approximation of the normalized cross-power spectrum matrix in the presence of corrupted components; and (3) a stable robust estimation algorithm is employed to effectively eliminate the residual outliers during line fitting. In addition, a fine registration method on the basis of the enhanced subpixel PC method is introduced, which is able to reduce the local misalignments between multisensor and multisource remote sensing images. The experiments carried out on remote sensing images from different satellites demonstrated the feasibility and reliability of the proposed method. In summary, the main contributions of this paper are: (1) an accurate and robust subpixel PC method for translation estimation is proposed, additionally embedding phase congruency-based structural representation, robust masked rank-one matrix approximation and robust model fitting using higher than minimal subset sampling; and (2) based on the enhanced subpixel PC matching, an automatic and reliable fine registration method for multisensor remote sensing images is presented, combining with the block-based phase congruency feature detector and local warping model.

The remainder of this paper is organized as follows. Related work is briefly reviewed in Section 2. The details of the proposed subpixel PC method and fine registration method are described in Section 3 and Section 4, respectively. Section 5 presents the experimental results and analysis, including the tie point matching experiment and fine registration experiment. Finally, the concluding remarks and considerations for future work are given in Section 6.

## 2. Related Work

### 2.1. Fine Registration Using Area-Based Methods

Area-based matching methods directly utilize intensity-based information to match images or regions. This type of matching method is widely used to optimize the coarse registration of remote sensing images due to the superiority in accuracy [24]. The adopted similarity measure is a decisive component of area-based methods. The conventional ones mainly include the sum of squared difference, the sum of absolute difference, the normalized cross correlation (NCC) [25], but are sensitive to nonlinear intensity changes [26]. In order to enhance the illumination robustness, several more sophisticated similarity measures such as mutual information (MI) [27], cross cumulative residual entropy [28], Jeffrey’s divergence [29], and matching by tone matching (MTM) [30] have been developed and broadly applied in remote sensing image registration [31,32]. In [33] and [34], MI-based metrics were utilized in optimization procedure to refine the coarse results of feature-based registration. In [35], normalized gradient field was adopted as a similarity measure to align the georeferenced airborne light detection and ranging (LiDAR), hyperspectral and photographic imagery. Moreover, some structure and shape features have been recently adopted as the replacement of image intensity and combined with the conventional similarity measure to reduce the influence of complicated radiometric difference on image registration [19,36]. The histogram of orientated phase congruency (HOPC) descriptor and the scene shape similarity feature descriptor were proposed in [9] and [37] respectively, and combined with NCC to achieve multimodal remote sensing image registration. In [38], a novel similarity measure was developed for optical-to-synthetic aperture radar (SAR) image matching as the NCC between dense rank-based local self-similarity descriptors. However, these similarity measures are somewhat computationally expensive or merely determine the subpixel measurement through simple polynomial fitting [39].

### 2.2. Phase Correlation

PC is a special area-based method calculated through frequency-domain operation. The theoretical basis of PC matching is the translation property of FT that links the shift of two relevant images in the spatial domain with the phase difference in the frequency domain. Assuming an image f(x,y) and another shifted image g(x,y)=f(x−Δx,y−Δy), the normalized cross-power spectrum can be calculated by [40]:

(1) $$Q(u,v)=\frac{{ℱ}_{f}{\left(u,v\right)}^{*}{ℱ}_{g}(u,v)}{\left|{ℱ}_{f}{\left(u,v\right)}^{*}{ℱ}_{g}(u,v)\right|}=\exp(-i(u\Delta x+v\Delta y))$$

where ${ℱ}_{f}(u,v)$ and ${ℱ}_{g}(u,v)$ are the corresponding frequency representations of two images after FT, *i* is the first solution to the equation $i^{2}=-1$, and * denotes the complex conjugate. The correlation function of PC is derived as the inverse FT of the normalized cross-power spectrum. In the ideal case of integer shifts, this correlation function corresponds to a Dirac delta function centered at (Δx,Δy). Accordingly, the pixel-level results of PC can be obtained by locating the peak of the correlation function.

In the case of subpixel shifts, the signal power of PC is not concentrated in a single peak, and leads to a downsampled 2-D Dirichlet kernel [40]. The existing subpixel PC methods can be found in two categories [20]. The first category is implemented in the spatial domain. The objective is to determine the fractional peak location of the correlation function with maximum correlation value, similar to the pixel-level matching. This can be achieved through similarity fitting with a certain set of neighbors using analytical derivations [40] or empirical fitting models [41], as well as upsampling the correlation function to a desired resolution in the frequency domain [42]. These methods have been successfully applied in the fine registration of multisensor remote sensing images [10,43], but they are vulnerable to the actual noise and aliasing.

The second category is realized in the frequency domain, which relies on the phase difference between two images, which is defined as the phase angle of the complex normalized cross-power spectrum. According to Euler’s formula, the phase difference can be expressed by:

(2) φ(u,v)=∠Q(u,v)=−(uΔx+vΔy).

It can be found that the phase difference is a linear function of the shift vector, and the shifts can thus be estimated from the slope of phase difference. In this case, subpixel PC methods in the frequency domain are calculated by plane fitting [44,45], line fitting [46,47], or nonlinear optimization [48] with the linear phase difference between images. Note that the phase difference is 2π wrapped when dealing with discrete image signals, and phase unwrapping is needed in practice when estimating the shifts greater than 0.5 pixels. Due to avoiding the inverse FT process and relying on a theoretical expression, the second category usually has advantages in matching accuracy and robustness over the first category [49].

## 3. Enhanced Subpixel Phase Correlation

### 3.1. Workflow of the Enhanced Subpixel Method

**Figure 1:**
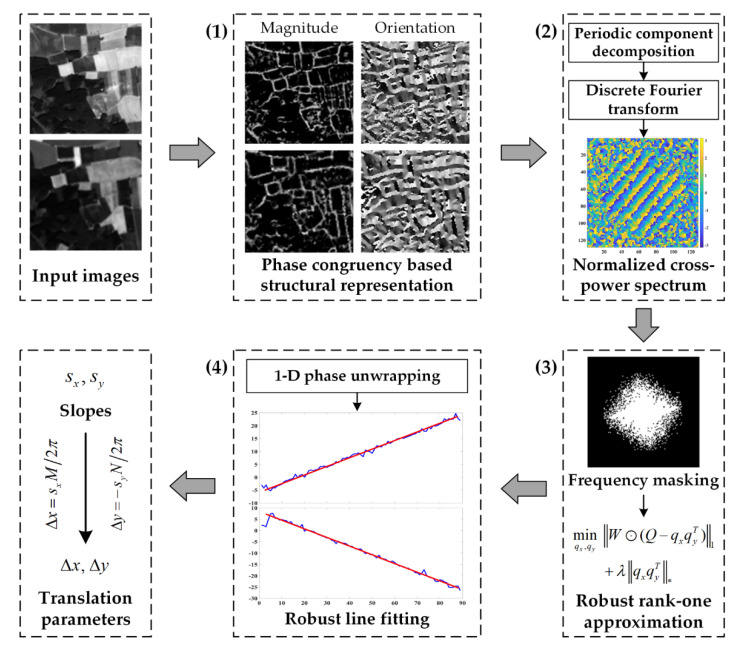
Overall workflow of the enhanced subpixel phase correlation method.

The proposed subpixel PC method calculates the translation parameters in the frequency domain by means of the phase difference between input images. The overall workflow of the proposed method, which mainly consists of four steps, is depicted in Figure 1 and introduced in the following.

(1) Construction of phase congruency-based structural representation. In order to minimize the influence of complicated intensity differences and emphasize the useful structural information for matching, we adopt the phase congruency [50] to generate a complex structural representation. The magnitude and orientation of the phase congruency features are combined to replace the original image intensity for the following image matching.
(2) Calculation of normalized cross-power spectrum. The structural representations are transferred to the frequency domain using discrete FT. However, the periodicity of discrete FT induces the edge effect that affects the performance of PC. Therefore, we use an image decomposition algorithm [51] to extract the periodic component to eliminate the edge effects. Compared with the conventional windowing operation, this decomposition avoids narrowing the effective matching region and loss of image information [52]. The normalized cross-power spectrum matrix *Q* is then calculated as Equation (1).
(3) Frequency masking and rank-one matrix approximation. In uncontrolled conditions, noise, aliasing, and other interference factors will contaminate the spectral components and degrade the following rank-one approximation and line fitting processing. In this case, we apply an adaptive frequency masking operation to filter out the corrupted frequency components [48]. Subsequently, two 1-D column vectors are factorized from the normalized cross-power spectrum matrix by determining the best rank-one approximation using a low-rank matrix approximation algorithm [53] which is robust to missing masked data and outliers.
(4) Estimation of translation parameters. With the low-rank vectors obtained, the phase difference is separately extracted in each dimension after 1-D phase unwrapping. The correct slopes $\left(s_{x},s_{y}\right)$ of the unwrapped phase angles are identified by a robust estimation algorithm using higher than minimal subset sampling [54] in the presence of residual outliers, and finally converted to the results of translation parameters according to $\Delta x=s_{x}M/2\pi$, $\Delta y=-s_{y}N/2\pi$, where *M* and *N* denote the size of the input images.

### 3.2. Details of the Enhanced Subpixel Method

To ensure the high accuracy and robustness, the enhanced subpixel PC method additionally integrates phase congruency-based structural representation, robust rank-one matrix approximation with adaptive frequency masking, and stable robust line fitting. All of these operations aim to guarantee that the practical phase difference calculated in tie point matching better agrees with the theoretical model in Equation (2).

### 3.2.1. Phase Congruency-Based Structural Representation

Although PC is insensitive to image content and intensity changes to some extent since it relates solely to phase information, the complicated radiometric changes can still deteriorate the linear relationship of the phase difference between input images [55]. The illumination robustness can be improved by constructing a structural representation combining the magnitude and orientation of phase congruency [56]. Phase congruency is a feature measure based on local frequency analysis, which perceives the corner and edge features where the Fourier components are maximal in phase. Phase congruency conforms to the human visual perception of image features, and has been widely applied in multimodal registration and matching [9,11,36]. By convolving a 2-D image f(x,y) through log-Gabor filters over several scales and orientations, the magnitude $A_{no}$ and phase $\phi_{no}$ of the filter responses at a scale *n* and orientation *o* are given by:

(3) $$\begin{array}{l} A_{n}=\sqrt{e_{n}{\left(x,y\right)}^{2}+o_{n}{\left(x,y\right)}^{2}}\\ \phi_{n}=\mathrm{atan}2(e_{n}(x,y),o_{n}(x,y))\\ \left[e_{n}(x,y),o_{n}(x,y)\right]=\left[f(x,y)*M_{n}^{e},f(x,y)*M_{n}^{o}\right] \end{array}$$

where $M_{n}^{e}$ and $M_{n}^{o}$ denote the log Gabor even-symmetric and odd-symmetric wavelets that are the real and imaginary components of log-Gabor filters, respectively, $e_{no}$ and $o_{no}$ denote the convolution results of these two wavelets. The magnitude of the phase congruency can be expressed as [50]:

(4) $$\begin{array}{l} PC(x,y)=\frac{\sum_{o}\sum_{n}W_{o}(x,y)\lfloor A_{no}(x,y)\Delta \Phi_{no}(x,y)-T\rfloor }{\sum_{o}\sum_{n}A_{no}(x,y)+\varepsilon }\\ \Delta \Phi_{no}(x,y)=\cos(\phi_{no}(x,y)-\bar{\phi }(x,y))-\left|\sin(\phi_{no}(x,y)-\bar{\phi }(x,y))\right| \end{array}$$

where $\bar{\phi }$ is the mean phase, *W* is a weighting term based on the frequency spread, *T* is a noise threshold, ε is a small constant and the symbol ⌊⌋ denotes that the enclosed quantity is equal to itself when its value is positive, or is zero otherwise. The orientation of the phase congruency can be calculated using the log Gabor odd-symmetric wavelets of multiple directions, which is expressed as:

(5) $$\Phi (x,y)=\mathrm{atan}2(\sum_{\theta }\left(o_{no}(x,y)\sin(\theta )\right),\sum_{\theta }\left(o_{no}(x,y)\cos(\theta )\right)),$$

where θ is the orientation angle. Then, the phase congruency-based structural representation is constructed as:

(6) $$R_{PC}(x,y)=PC(x,y)\cos(\Phi (x,y))+iPC(x,y)\sin(\Phi (x,y)).$$

The following subpixel PC is performed on the complex structural representations of both images instead of the original intensity. Both phase congruency and PC matching take advantage of the phase information of the image and are independent of magnitude information. Phase congruency relies on the local phase of images to preserve local topological information, while PC matching relies on the global phase difference to estimate the translation and similarity between images. Therefore, PC matching with phase congruency-based representations combines the global and local phase information to underline the frequency response of structural features and improve the robustness to local radiometric differences for translation estimation.

### 3.2.2. Robust Rank-One Matrix Approximation with Adaptive Frequency Masking

According to the expression in Equation (1), the normalized cross-power spectrum matrix *Q* is theoretically a rank-one matrix [46], i.e., $Q=q_{x}q_{y}^{T}$, where *q_x_* and *q_y_* are complex column vectors. This implies that the 2-D translation estimation can be converted to two separate 1-D problems by finding the dominant rank-one subspace of *Q*. The most straightforward way is to use the singular value decomposition algorithm. However, the corrupted spectral components caused by noise, aliasing, and other interference factors in practice will potentially bias the ideal rank-one computation and the final estimation results [48,57]. Therefore, an effective frequency masking operation to remove the corrupted components and a robust low-rank matrix approximation algorithm to deal with missing data and outliers are adopted.

Since the high frequencies and the frequencies with small spectral magnitude that are most likely to be corrupted, the masking operation firstly masks out the high-frequency components at each periphery (e.g., 15% as suggested in [44]) of *Q*. Then, the unreliable frequency components with small magnitude are identified according to the normalized log-spectrum [48]. Therefore, the frequency mask is defined as:

(7) $$\begin{array}{l} W(u,v)=\left\{ \begin{array}{l} 0,\;\;u<0.15M;u>0.85M;v<0.15N;v>0.85N\\ 0,\;\;\mathrm{NLS}(u,v)\leq p\cdot \mathrm{mean}\{\mathrm{NLS}(u,v)\}\\ 1,\;\;\mathrm{others} \end{array}\right.\\ \mathrm{LS}(u,v)=\log_{10}\left|{ℱ}_{f}{\left(u,v\right)}^{*}{ℱ}_{g}(u,v)\right|\\ \mathrm{NLS}(u,v)=\mathrm{LS}(u,v)-\max\{\mathrm{LS}(u,v)\} \end{array}$$

where *p* is a specific parameter, we fix *p* = 0.9 the same as [48] for all the experiments.

The robust rank-one approximation is formulated as an optimization problem based on *L*_1_-norm loss and nuclear-norm regularizer [53], which is able to effectively handle the masked data and residual outliers. The objective function is written as:

(8) $$\min_{q_{x},q_{y}}{\left\|W⊙(Q-q_{x}q_{y}^{T})\right\|}_{1}+\lambda {\left\|q_{x}q_{y}^{T}\right\|}_{*},$$

where λ is a balancing parameter, *W* is the frequency masking matrix, the operator ⊙ denotes the element-by-element matrix product, the symbol ${\left\|\right\|}_{1}$ denotes *L*_1_-norm, and ${\left\|\right\|}_{*}$ denotes nuclear-norm which is defined as the sum of singular values. The regularized optimization problem can be solved by an augmented Lagrange multiplier method. By introducing a matrix $E=q_{x}q_{y}^{T}$ and some constraints, Equation (8) becomes:

(9) $$\begin{array}{l} \underset{E,q_{x},q_{y}}{\min}{\left\|W⊙(Q-E)\right\|}_{1}+\lambda {\left\|q_{y}^{T}\right\|}_{*}\\ s.t.,\;\;E=q_{x}q_{y}^{T},q_{x}^{T}q_{x}=1 \end{array}$$

The unconstrained augmented Lagrange function after adding a penalty term and a Lagrange multiplier *L* is given by:

(10) $$f(E,q_{x},q_{y},L,\mu )={\left\|W⊙(Q-E)\right\|}_{1}+\lambda {\left\|q_{y}^{T}\right\|}_{*}+\frac{\mu }{2}{\left\|E-q_{x}q_{y}^{T}\right\|}_{F}^{2}+\langle L,E-q_{x}q_{y}^{T}\rangle ,$$

where μ is a penalty parameter, the symbol ${\left\|\right\|}_{F}$ denotes Frobenius norm, and 〈A,B〉 is equivalent to the trace of $A^{T}B$. The complex column vectors can be solved by Gauss-Seidel iteration that iteratively solve one set of variables in $E,q_{x},q_{y}$ while fixing the other two with the Lagrange multiplier *L* and the penalty parameter μ updated in each iteration. More details of the optimization and implementation settings can be found in [53].

### 3.2.3. Stable Robust Line Fitting

To automatically exclude the corrupted phase angle values when fitting the slopes of the unwrapped phase angle vectors, a robust estimation algorithm using higher than minimal subset sampling (HMSS) [54] is introduced. Compared with the conventional random sample consensus algorithm [58], HMSS has two refinements: (1) it increases the initial sampling size beyond the minimal size to ensure the closeness of the hypothesis generation to the true model; (2) it is not a pure random sampling strategy, but a greedy strategy that starts from a random hypothesis and is iterated towards an optimized solution using the least *k*-th order statistic cost function until a stopping criterion is reached. These enable HMSS to achieve advantages on stability, accuracy, computational efficiency, and parameter insensitivity. The routine of our HMSS fitting is presented as follows. (1) For 2-D line fitting, five points (minimal size three plus two) from the unwrapped phase angle vectors are randomly selected to generate an initial model using a least-squares fitting. (2) In each iteration *l*, the residuals of all points are calculated and sorted, and the least *k*-th order statistic is calculated as a cost function:

(11) $$F(\delta_{l})=r_{i_{k,\delta_{l}}}^{2}(\delta_{l}),$$

where $r_{i}^{2}(\delta )$ denotes the *i*-th squared residual regarding model δ, $i_{k,\delta }$ denotes the index of *k*-th sorted squared residual regarding model δ, and *k* is an inlier parameter fixed at k=0.2⋅TN, where *TN* is the total data number. The model of next iteration $\delta_{l+1}$ is updated using the new five sample points around the *k*-th sorted square residual. (3) Iterations are continued until reaching a stopping criterion. The criterion is designed to check if the samples selected in consecutive iterations are from similar models, and is given by:

(12) $$F_{stop}=\left(r_{i_{k,\delta_{l}}}^{2}(\delta_{l})<\frac{1}{5}\sum_{j=k-5+1}^{k}r_{i_{j,\delta_{l-1}}}^{2}(\delta_{l})\right)\wedge \left(r_{i_{k,\delta_{l}}}^{2}(\delta_{l})<\frac{1}{5}\sum_{j=k-5+1}^{k}r_{i_{j,\delta_{l-2}}}^{2}(\delta_{l})\right),$$

where $r_{i_{j,\delta_{l-1}}}^{2}(\delta_{l})$ and $r_{i_{j,\delta_{l-2}}}^{2}(\delta_{l})$ denote the residuals of the sample points selected in two previous iterations with regard to the model $\delta_{l}$ in iteration *l*. If the current cost function is lower than the average residuals of those sample points, the sample points selected in the last three iterations are likely to belong to the same structure and the iteration can stop. (4) To decrease the probability of accident erroneous estimation, steps (1)–(3) are repeated for reinitialization of random hypothesis generation until there is no decreasing of the cost function in consecutive runs. (5) The model with the minimal cost function is selected and refined using all the inliers by least-squares method, and the algorithm output the final slope.

## 4. Multisensor Fine Registration

**Figure 2:**
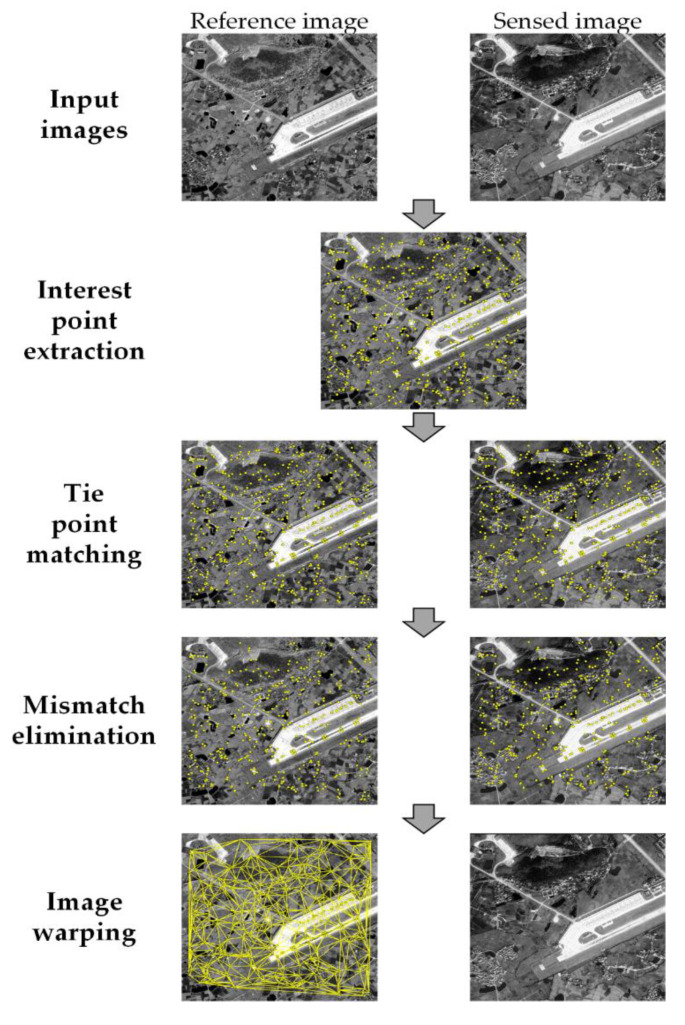
Flowchart of the fine registration method based on the enhanced phase correlation (PC) matching.

The enhanced subpixel PC method can provide accurate and robust translation estimation as local template matching. In this section, an automatic registration method for precisely aligning coarsely coregistered remote sensing images from different sensors is extended based on the enhanced subpixel PC method. The flowchart of the fine registration method is illustrated in Figure 2, which is divided into four steps as follows.

(1) Interest point extraction. To improve the localization performance in the presence of complicated radiometric conditions, phase-congruency corner detector is applied to detect the interest points on the reference image. According to Equation (4), we can obtain a phase congruency map. The moment analysis is performed on the phase congruency maps with different orientations, and the minimum moment *m* is given by [59]:
  (13) $$\begin{array}{l} m=\frac{1}{2}\left(c+a-\sqrt{b^{2}+{\left(a-c\right)}^{2}}\right)\\ a={\sum_{o}\left(PC(\theta )\cos(\theta )\right)}^{2}\\ b=2\sum_{o}\left(PC(\theta )\cos(\theta ))(PC(\theta )\sin(\theta )\right)\\ c={\sum_{o}\left(PC(\theta )\sin(\theta )\right)}^{2} \end{array}$$
  where PC(θ) is the phase congruency value determined at orientation angle θ. The minimum moment is equivalent to the cornerness measure. In order to extract the interest points uniformly distributed over the scene, a block-based strategy is adopted [19]. The image is partitioned into *s* × *s* nonoverlapping blocks, and the top *h* points with the largest minimum moment values are regarded as the interest points for each block.
(2) Tie point matching. The corresponding points on the sensed image are determined by PC-based template matching. A template window is selected surrounding each interest point. The translation parameters between template windows are estimated by the pixel-level PC matching and then refined using the enhanced subpixel PC method. Note that the phase congruency calculated in the last step can be reused in the subpixel PC matching.
(3) Mismatch elimination. There inevitably exist false matches in the results of tie point matching due to shadow and featureless areas. These mismatched tie points can be filtered by considering two aspects: the similarity measure and geometric consistency. The peak value of PC function provides a measure to assess the correctness of the match. The unreliable measurements with small PC peak values are firstly removed. Then, the residual outliers are eliminated by an iterative consistency check of tie points based on a global transformation [19]. In each iteration of consistency check, a transformation model is estimated using all the tie points with the transformation residuals calculated. The tie point with the largest residual is excluded, and the transformation model is estimated again on the remaining points. The procedure is repeated until the largest residual is less than a given threshold (e.g., 1.5 pixels). The three-order polynomial model is selected in this study since it can better handle the local deformations resulted from sensor error and terrain relief especially for high-resolution images.
(4) Image warping. With the refined tie points, a transformation model that maps the sensed image to the reference image can be determined. We employ a piecewise linear model that is known to be appropriate for mitigating local geometric distortions between satellite images [60]. This function divides the image into triangular regions by the Delaunay’s triangulation algorithm using all the tie points, and estimates an affine transformation for each triangular region. For warping the regions outside the convex hull of the points, we estimate a global transformation model from the points defining the convex hull [61].

## 5. Experiments and Discussion

In order to verify the effectiveness of the proposed method, experiments were conducted in two parts, a tie point matching experiment and a fine registration experiment. The tie point matching experiment evaluates the matching performance of the enhanced subpixel PC method, and the fine registration experiment analyzes the alignment performance of the presented registration method based on the enhanced PC matching.

### 5.1. Tie Point Matching Experiment

#### 5.1.1. Experimental Details

In this experiment, the enhanced subpixel PC method was assessed and compared with other PC methods and area-based matching methods. The block-based phase congruency detector was first applied to extract 400 interest points (top four points in each 10 × 10 nonoverlapping blocks) uniformly distributed over the reference image, whose corresponding points were then determined by template matching. The results obtained from the proposed method were compared with those from five state-of-the-art Fourier-based correlation methods including PC with quadratic fitting (PC_QF), Foroosh’s method [40], upsampled cross correlation (UCC) [42], Hoge’s method [46], and SVD-RANSAC (singular value decomposition-random sample consensus) [49], as well as five other representative area-based matching methods including NCC [25], MI [27], MTM [30], HOPCncc (NCC of the HOPC descriptors) [9] and enhanced correlation coefficient (ECC) [62]. PC_QF, NCC and MI are available in MATLAB; the codes of UCC, MTM, HOPCncc, and ECC are provided by the authors, and the others are our re-implementations. For the Fourier-based correlation methods, the image decomposition algorithm was adopted to mitigate the influence of edge effects. For PC_QF, NCC, MTM, and HOPCncc, the subpixel measurements were obtained by fitting the similarity function using a quadratic polynomial model. Three sizes of template windows (40 × 40, 60 × 60, 80 × 80 pixels) were tested to analyze the matching performance under different template sizes, and the size of search region was set as 20 × 20 pixels.

Three sets of remote sensing image pairs acquired from different satellites and sensors were used. The basic information about these multisensor images are given in Table 1. Each image pair contains a reference image (upper) and a sensed image (lower) captured by different sensors with diverse spatial resolution and imaging data. All these image pairs have been coarsely registered based on the metadata and georeferencing information, and resampled to the same ground sampling distance. For the image pair with large deviation due to the sensor positioning error (e.g., approximate 70 pixels for Data 1), the global translations between images were compensated using the pixel-level PC with inputs of the entire image. Therefore, the test image pairs are free of obvious scale, rotation, and translation differences, but still show significant intensity and textural changes due to various resolution, imaging time, and spectrum.

For each test data, 40–50 evenly distributed check points were manually selected from the reference and sensed images, and a three-order polynomial model can be estimated from the check points. The matching errors of tie points were then measured according to this transformation model, and the correct matches were identified as the tie points with matching errors smaller than a threshold. This threshold was set as 1 pixel for Data 2 and Data 3, and set as 1.5 pixels for Data 1 because of more severe local distortions and higher spatial resolution. The evaluation criteria used in this experiment include the precision and root mean square error (RMSE) of tie points. The precision refers to the correct match rate calculated as the number of correct matches divided by the total number of matches. The RMSE between transformed points and matched points was calculated from both the correct matches and total matches to evaluate the matching accuracy and stability.

#### 5.1.2. Results and Discussion

**Figure 3:**
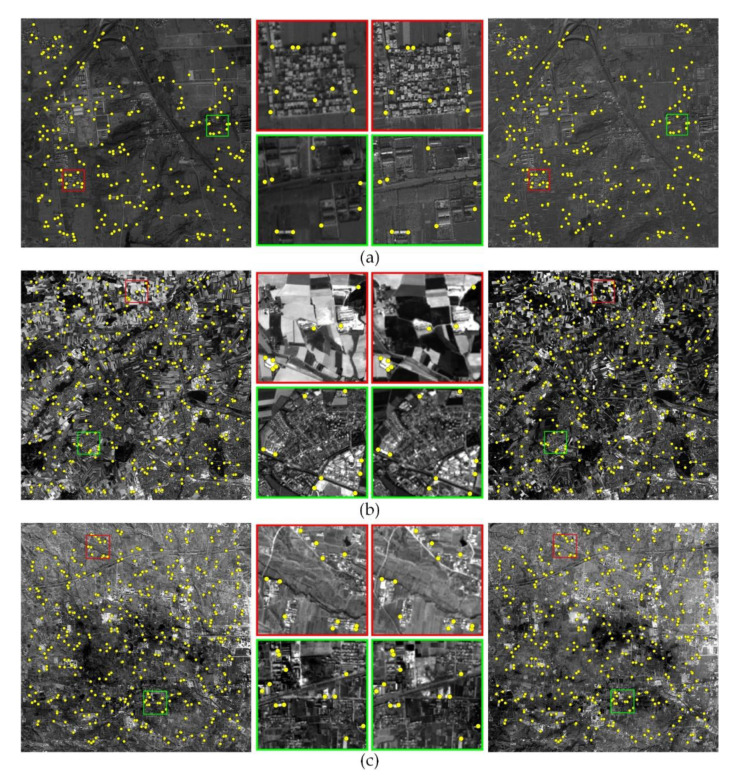
Tie points achieved by the enhanced subpixel PC method with the template size of 80 × 80 pixels for three test image pair. (**a**) Data 1; (**b**) Data 2; and (**c**) Data 3.

Figure 3 displays the tie points achieved by the block-based phase congruency detector and the enhanced subpixel PC method. It can be seen that the image pair represents significant radiometric and textural differences. The enhanced subpixel PC method is able to identify enough well-distributed tie points in multisensor remote sensing images, and the locations of tie points correctly correspond to each other. This will be beneficial for the following multisensor fine registration.

Since the proposed method embeds three additional operations, first the performance gain of each individual operation was demonstrated. Besides the baseline of Hoge’s method and the final proposed method, two variants were also evaluated using Data 1: Variant 1 combines Hoge’s method with structural representation; Variant 2 combines Variant 1 with robust model fitting; and the final proposed method further embeds robust masked rank-one matrix approximation. The precisions and RMSEs of the total matches of these four methods are shown in Table 2. It can be seen that the matching performance gradually improves from the baseline method to Variant 1, Variant 2, and the final proposed method by integrating different additional operation. This indicates that the phase congruency-based structural representation, robust masked rank-one matrix approximation and stable robust model fitting are all effective to enhance the matching accuracy and robustness.

**Figure 4:**
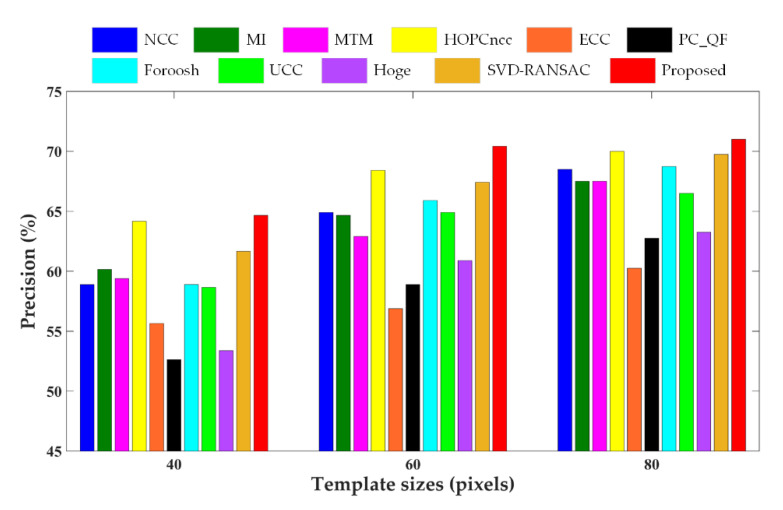
Precision values of different methods for Data 1. NCC, normalized cross correlation; MI, mutual information; MTM, matching by tone matching; HOPCncc, NCC of the HOPC descriptors; ECC, enhanced correlation coefficient; PC_QF, PC with quadratic fitting; UCC, upsampled cross correlation; SVD-RANSAC, singular value decomposition-random sample consensus.

**Figure 5:**
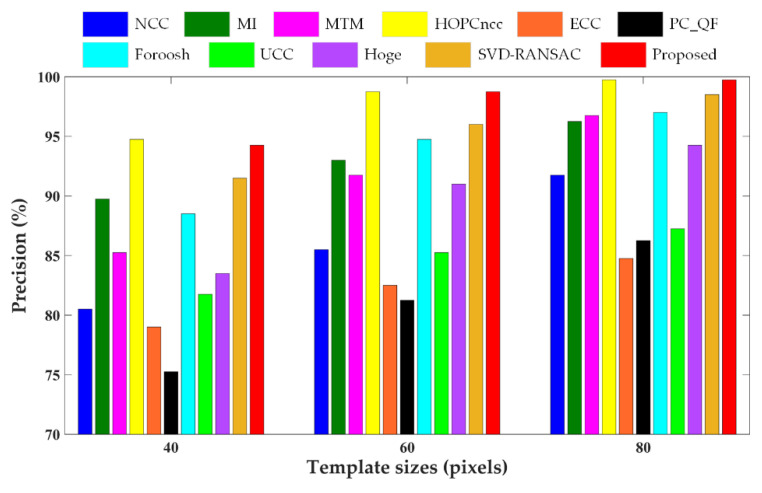
Precision values of different methods for Data 2.

**Figure 6:**
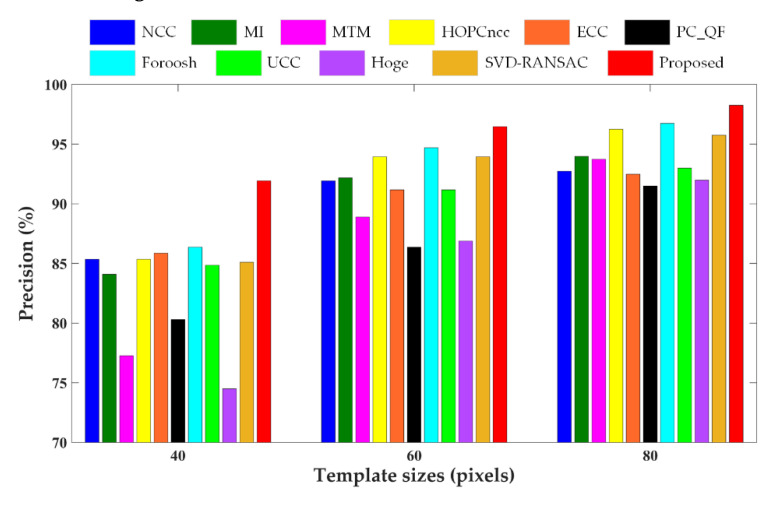
Precision values of different methods for Data 3.

The comparative results of different template matching methods in terms of matching precision are shown in Figure 4, Figure 5 and Figure 6 for three test data, respectively, and the RMSEs of the correct matches and total matches of various matching methods with three different template sizes are presented in Table 3. As can be seen from the figures and table, the enhanced subpixel PC method, SVD-RANSAC, and HOPCncc generate the overall best results, achieving higher values of matching precision and lower RMSEs. The performances of other methods are negatively affected by the complicated radiometric and textural changes in multisensor images, which are manifested by more false matches and inferior matching accuracy in the results. With regard to the RMSEs of the correct matches, the proposed method reaches the lowest values for Data 2 and 3, but is not obviously better for Data 1. The possible explanation for Data 1 is due to the severe local distortions. Since the correct matches are identified using the manual check points by thresholding, the RMSEs of the correct matches will be close to the accuracy of check points in the case of severe local distortions, and are less dominated by the accuracy of matching algorithms. Compared with other line fitting-based PC methods, such as Hoge’s, SVD-RANSAC, and other Fourier-based correlation methods, the proposed method improves the accuracy and robustness of subpixel translation estimation by integrating phase congruency-based structural representation, *L*_1_-norm-based rank-one matrix approximation with frequency masking and robust model fitting using higher than minimal subset sampling. Based on the resistance of phase congruency to nonlinear intensity difference [9], HOPCncc obtains the comparable results in most cases. In general, the proposed method performs better than HOPCncc method. The improved correct match rate and subpixel capability benefit from the use of pixelwise structure representation and theoretical model based on the translation property of FT. The experimental results demonstrate the superiority and reliability of the proposed method in tie point matching of multisensor remote sensing images.

It can be found that the matching performance of all methods is related to the template sizes. The matching precision and accuracy become worse with the decreasing template sizes due to less structural information for matching. Frequency-based image correlation methods are hypothesized to be more limited in small template sizes following the Heisenberg’s uncertainty principle [20]. In this case, several obviously erroneous measurements exist in the matching results that affect the RMSEs. In addition, the local geometric distortions degrade the matching results. For Data 1 with higher resolution and larger geo-positioning errors, the success match ratio is significantly lower than for the other two datasets. Therefore, a potential refinement point of the proposed method in future work is to mitigate the influence of local geometric deformations, especially in the case of a small template.

### 5.2. Fine Registration Experiment

#### 5.2.1. Experimental Details

In this experiment, the fine registration method presented was validated and compared with feature-based methods. Besides the above-mentioned HOPCncc and SVD-RANSAC methods, three state-of-the-art feature detectors and descriptors, namely SIFT [14], ORB (oriented features from accelerated segment test and rotated binary robust independent elementary features) [63], and RIFT (radiation-variation insensitive feature transform) [59] were used for comparison. SIFT detects keypoints based on the Difference-of-Gaussian scale space and generates a float-type descriptor for each feature based on the orientation of image gradient. ORB identifies keypoints using an oriented version of FAST corner detector and computes a binary-type feature vector using the rotation-aware BRIEF descriptor. RIFT extracts radiation-robust features based on phase congruency and log-Gabor convolution of different orientations. For the feature-based methods, the nearest neighbor distance ratio strategy [14] and random sample consensus algorithm [58] were adopted to eliminate the outliers in the matched features. For the area-based methods, the fine registration pipeline introduced in Section 4 was adopted, wherein a set of 600–700 evenly distributed tie points were extracted and matched based on the block-based phase congruency detector and the corresponding template matching methods. The piecewise linear transformation model was employed to warp the sensed image according to the tie points obtained by different methods.

As shown in Table 4, three sets of multisensor optical image pairs were tested in this experiment. These images range from very high resolution (submeter) to medium resolution (dozen of meters), and cover different scenes including urban and suburban areas. A temporal difference also exists between reference and sensed images with the maximum gap for more than three years. Similarity, all these image pairs have been preregistered though direct georeferencing and resampled to the same resolution of reference image to remove the obvious rotation, scale, and translation differences.

The distribution quality of tie points and final registration performance were assessed for all four methods. The distribution quality was measured by an index considering the area and shape of the triangles formed by tie points [64], which can be defined as:

(14) $$\begin{array}{l} D_{A}=\sqrt{\frac{\sum_{i=1}^{t}{\left(\frac{A_{i}}{\bar{A}}-1\right)}^{2}}{t-1}},\;\;\bar{A}=\frac{\sum_{i=1}^{t}A_{i}}{t}\\ D_{S}=\sqrt{\frac{\sum_{i=1}^{t}{\left(S_{i}-1\right)}^{2}}{t-1}},\;\;S_{i}=\frac{3\cdot \max(J_{i})}{\pi }\\ DQ=D_{A}\cdot D_{S}=\frac{\sqrt{\sum_{i=1}^{t}{\left(\frac{A_{i}}{\bar{A}}-1\right)}^{2}\cdot \sum_{i=1}^{t}{\left(S_{i}-1\right)}^{2}}}{t-1} \end{array}$$

where *t* is the number of triangles, $A_{i}$ denotes the area of the *i*-th triangle, and $\max(J_{i})$ denotes the largest internal angle of the *i*-th triangle. The smaller value of DQ indicates in Equation (14) the better distribution of tie points. To evaluate the quantitative registration performance, 40–50 evenly distributed check points were manually selected between each image pair, and the RMSE and standard deviation (STD) of the check points after registration was calculated.

#### 5.2.2. Results and Discussion

**Figure 7:**
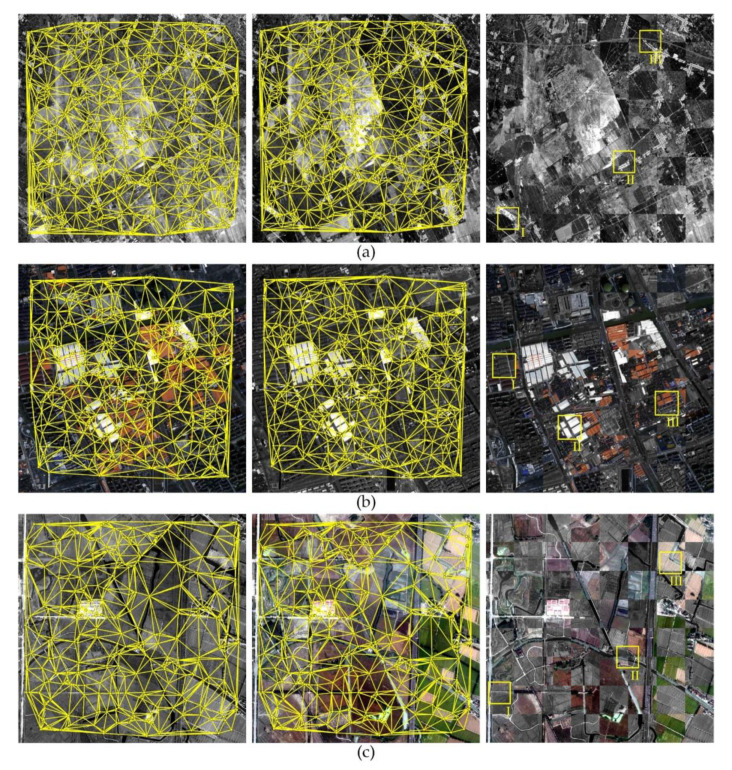
Registration results of the presented method for three test image pair. (**a**) Data 1; (**b**) Data 2; and (**c**) Data 3.

**Figure 8:**
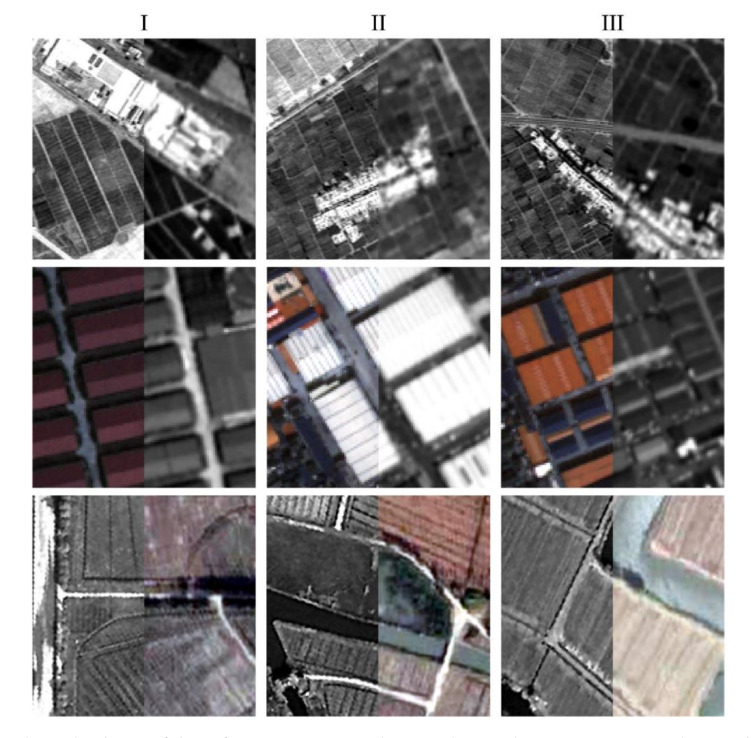
Enlarged subsets of the reference images and warped sensed images corresponding to the boxes **I**, **II**, and **III** in Figure 7.

In our registration method, the tie points were matched by the enhanced subpixel PC and filtered by the correlation values and iterative consistency check, and the warped sensed images were generated by the combination of piecewise linear functions and a global transformation. The registration results, including the Delaunay triangulations constructed from the filtered tie points and the chessboard images generated from the reference images and warped sensed images, are shown in Figure 7, and the enlarged subsets corresponding to the sample regions in the third column of Figure 7 are presented in Figure 8. It can be seen that the scenes accord well in two images after fine registration for all test cases with a simple visual inspection of the registration results, which qualitatively confirms the satisfactory registration performance of the presented method based on the enhanced PC matching.

In order to further validate the effectiveness, the number of matches, distribution quality index, RMSE, and STD of check points obtained from different methods are reported in Table 5. From the comparative results, it can be seen that the registration method presented significantly outperforms the other three feature-based methods in terms of distribution quality and registration accuracy. The presented method obtains tie points with the minimum value of DQ index indicating better distribution over the entire image. This is attributed to adopting the block-based strategy and limiting the search range, which is one of the advantages of area-based matching methods. The excellent distribution and high matching accuracy of tie points facilitate a good registration using a nonrigid piecewise linear model. Therefore, the presented method achieves a higher and more uniform registration accuracy with the minimum values of both RMSE and STD in the numerical analysis compared with the other three feature-based methods. Moreover, the presented method using the enhanced subpixel PC matching also obtains consistently lower values of RMSE and STD than the HOPCncc and SVD-RANSAC methods. It is worth noting that the RMSEs of three test data are all less than 1 pixel for our registration method, but grow with the decreasing spatial resolutions. The qualitative and quantitative analyses indicate that the presented method has the capability to offer an automatic and reliable solution to fine registration of multisensor remote sensing images.

## 6. Conclusions

In this paper, we propose an enhanced subpixel PC method and perform fine registration of multisensor remote sensing images based on this subpixel PC matching. The enhanced subpixel PC method achieves accurate and reliable template matching by adopting phase congruency-based structural representation, *L*_1_-norm-based rank-one matrix approximation with masking data, and stable robust model fitting. These operations ensure the calculated phase difference in practice better agree with the theoretical linear model based on the translation property of FT. The fine registration method combines the enhanced subpixel PC matching with block-based phase congruency feature detector, iterative consistency check, and image warping using piecewise linear transformation to precisely coregister the images from different satellites and sensors. Tie point matching and fine registration experiments were conducted, each using three sets of multisensor image pairs. In the tie point matching experiment, the enhanced subpixel PC method outperformed other state-of-the-art PC and area-based methods with a higher correct match rate and better matching accuracy. In the fine registration experiment, the proposed fine registration method outperformed state-of-the-art feature-based methods in terms of distribution quality and registration performance. The promising results indicate that the proposed method is robust and effective for multisensor fine registration.

Local deformation is an impact factor degrading the matching performance. The proposed method may be less effective in the presence of severe relief displacements, which is a common issue for high-resolution image registration. In future work, the proposed method will be refined to mitigate the influence of local deformation and utilize the prior knowledge from digital surface model and shadow map. In addition, this study mainly presents fine registration of multisensor optical remote sensing images, future works will explore the performance in more complicated multimodal applications.

## Figures and Tables

**Table 1:** Basic information about the images used in the tie point matching experiment.

Data No.	Image Sources	Size	Sensor Resolution	Date	Location
1	ZiYuan-3 PAN	1920 × 1980	2.1 m	2012/02	Dengfeng, Henan, China
	THEOS PAN	1990 × 1992	2 m	2011/12	
2	Sentinel-2 MSI Band 3	1800 × 1800	10 m	2015/08	Munich, Germany
	Landsat 8 OLI Band 8	1805 × 1805	15 m	2014/06	
3	Mapping-1 PAN	1720 × 1720	5 m	2013/05	Dengfeng, Henan, China
	ZiYuan-3 MUX Band 3	1725 × 1725	5.8 m	2012/02	

**Table 2:** Matching performance of the baseline, two variants and the proposed method (root mean square error (RMSE) unit: pixels).

Criterion		Hoge	Variant 1	Variant 2	Proposed
40	Precision	53.38%	56.14%	63.16%	64.66%
	RMSE	2.928	2.631	2.245	2.147
60	Precision	60.9%	63.91%	68.92%	70.43%
	RMSE	2.436	2.193	1.813	1.693
80	Precision	63.25%	66%	70%	71%
	RMSE	2.032	1.912	1.641	1.607

**Table 3:** RMSEs of the correct matches (CM) and total matches (TM) of various matching methods with three different template sizes (unit: pixels).

No.	Template Size		NCC	MI	MTM	HOPCncc	ECC	PC_QF	Foroosh	UCC	Hoge	SVD-RANSAC	Proposed
Data 1	40	CM	0.756	0.775	0.762	0.788	0.759	0.813	0.800	0.749	0.802	0.783	0.775
		TM	3.477	3.705	3.446	2.266	4.344	3.695	4.408	4.421	2.928	2.379	2.147
	60	CM	0.754	0.738	0.732	0.750	0.787	0.783	0.790	0.749	0.765	0.750	0.755
		TM	2.977	2.907	2.256	1.842	4.135	2.811	3.036	3.555	2.436	1.825	1.693
	80	CM	0.780	0.735	0.743	0.752	0.782	0.757	0.765	0.752	0.766	0.753	0.748
		TM	2.247	2.186	2.146	1.591	3.652	2.483	2.158	2.988	2.032	1.659	1.607
Data 2	40	CM	0.404	0.429	0.408	0.424	0.406	0.431	0.465	0.391	0.376	0.392	0.385
		TM	3.435	2.970	2.597	0.732	3.903	2.340	2.649	3.875	1.808	0.914	0.822
	60	CM	0.405	0.409	0.401	0.414	0.413	0.405	0.462	0.383	0.371	0.377	0.369
		TM	3.154	2.752	1.711	0.461	3.352	2.103	1.846	3.711	1.497	0.744	0.558
	80	CM	0.388	0.401	0.396	0.377	0.407	0.397	0.460	0.363	0.359	0.359	0.350
		TM	2.418	1.998	1.307	0.383	3.205	1.977	1.309	3.587	0.942	0.457	0.358
Data 3	40	CM	0.425	0.469	0.466	0.492	0.447	0.456	0.450	0.421	0.409	0.434	0.389
		TM	1.608	1.983	3.062	0.998	1.619	2.540	2.631	2.345	2.113	1.760	1.218
	60	CM	0.408	0.427	0.410	0.459	0.400	0.442	0.430	0.395	0.380	0.409	0.381
		TM	1.044	0.977	1.603	0.586	1.127	1.920	1.091	1.524	1.215	0.685	0.702
	80	CM	0.380	0.414	0.387	0.442	0.386	0.418	0.415	0.376	0.374	0.382	0.361
		TM	0.671	0.566	0.543	0.498	1.054	1.201	0.512	1.248	0.874	0.470	0.396

**Table 4:** Basic information about the images used in the fine registration experiment.

Data No.	Image Sources	Size	Sensor Resolution	Date	Location
1	SPOT-5 PAN	1750 × 1700	5 m	2013/06	Zhangye, Gansu, China
	Sentinel-2 MSI Band 3	1791 × 1716	10 m	2015/08	
2	GeoEye-1 RGB	1040 × 1010	2 m	2010/02	Shanghai, China
	ZiYuan-3 PAN	1044 × 1011	2.1 m	2013/07	
3	Hongqi-1-H9 PAN	2120 × 2140	0.75 m	2020/02	Shanghai, China
	Google earth	2124 × 2148	1.19 m	2019/10	

**Table 5:** Registration performance of different methods. TN, the number of total matches; RN, the number after outlier removal. The unit of RMSE and STD is pixels.

No.	Criterion	SIFT	ORB	RIFT	HOPCncc	SVD-RANSAC	Proposed
Data 1	RN/TN	1538/2689	1312/2121	502/1403	669/711	657/711	662/711
	DQ	2.648	3.911	1.4231	0.841	0.855	0.852
	RMSE	0.918	0.898	1.227	0.527	0.520	0.494
	STD	0.471	0.422	0.571	0.290	0.284	0.272
Data 2	RN/TN	178/865	456/1306	332/1040	498/600	486/600	495/600
	DQ	1.684	2.437	1.083	0.882	0.816	0.821
	RMSE	1.361	1.480	1.220	0.691	0.686	0.642
	STD	0.695	0.797	0.572	0.341	0.347	0.330
Data 3	RN/TN	56/849	67/565	165/907	391/713	378/713	399/713
	DQ	1.652	2.127	1.167	1.152	1.196	1.131
	RMSE	1.894	1.822	1.538	0.771	0.809	0.711
	STD	0.709	0.837	0.695	0.375	0.366	0.351
